# Pseudotumor from Metal-on-Metal Total Hip Arthroplasty Causing Unilateral Leg Edema: Case Presentation and Literature Review

**DOI:** 10.1089/biores.2017.0035

**Published:** 2018-03-01

**Authors:** Caleb W. Grote, Paul C. Cowan, David W. Anderson, Kimberly J. Templeton

**Affiliations:** ^1^ Department of Orthopedic Surgery, University of Kansas Medical Center, Kansas City, Kansas.; ^2^ Department of Orthopedic Surgery, Drisko, Fee and Parkins, Kansas City, Missouri.; ^3^ Department of Orthopedic Surgery, Kansas City Joint Replacement at Menorah Medical Center, Overland Park, Kansas.

**Keywords:** metal-on-metal total hip arthroplasty, women's health, pseudotumor, unilateral leg edema

## Abstract

Metal-on-metal (MoM) total hip arthroplasty (THA) can be associated with adverse metal reactions, including pseudotumors. This case report describes a 58-year-old female with an MoM THA-related pseudotumor that caused unilateral leg edema from compression of her external iliac vein. After thorough preoperative workup to rule out infection and deep vein thrombosis and consultation with a vascular surgeon, the patient underwent revision THA and excision of her pseudotumor. She had complete resolution of her swelling at 4 years after surgery. Review of all available case reports for this rare complication revealed that almost all patients were female. All patients underwent revision THA, with resolution of their symptoms. Literature review demonstrates that women are disproportionally affected by complications associated with MoM THA. We recommend close monitoring of patients with MoM THA, particularly women, for development of adverse metal reactions.

## Introduction

Total hip arthroplasty (THA) has become a widely used treatment for alleviating pain and disability secondary to degenerative joint disease of the hip. Women are at increased risk of osteoarthritis than men.^1^ There are currently an estimated 2.5 million people in the United States living with a THA.^2^

Over 200,000 THAs are performed in the United States each year, and this number is expected to rise to nearly 570,000 by 2030.^3^ While many implant variations have been described, the overall design has remained constant. This consists of a femoral stem that is inserted into the proximal femur and a femoral head that is attached to the stem, which then articulates with a cup that is set into the native acetabulum.

Early implant designs involved a metal femoral head articulation with a “conventional” plastic (polyethylene) liner within a metal cup. Early “conventional” polyethylene was associated with higher rates of asymmetric wear, particle debris, osteolysis, and implant failure.^4^ To address these concerns, implants with superior wear characteristics were developed. In the late 1990s, gamma radiation and thermal treatments were used to create highly crosslinked polyethylene, which showed significant improvement over “conventional” polyethylene and is commonly used in implants today and considered the “gold standard.”^4^

To further address issues seen with the conventional metal-on-polyethylene hip replacements, other bearing surfaces, termed “hard-on-hard,” have been developed. These include ceramic-on-ceramic and metal-on-metal (MoM) designs. MoM designs are not new—they were first described in the 1960s but experienced significantly increased utilization in the late 1990s.^4,5^ MoM bearing surface constructs have a metal femoral head that articulates directly with a smooth metal acetabular cup, providing two theoretical advantages over metal-on-polyethylene designs. First, these systems eliminate the thickness of the polyethylene, allowing for larger head sizes and related increased stability.^5–7^ Second, the “hard-on-hard” bearing surfaces demonstrate less volumetric wear and smaller particle generation, which could potentially decrease osteolysis^5,7–9^ and related implant loosening.

However, review of large-scale registry data in the early 2000s showed that MoM implants have a significantly higher revision rate compared with other implants.^10–12^ This eventually led to the recall of several MoM THA constructs,^5,13^ and MoM THA implantation has declined from 20% of THAs in 2005 to <1% in 2012.^14^ The increased revision rate of MoM THA is thought to be associated with the development of unique complications now known as adverse reactions to metal debris (ARMDs), which include elevated serum metal ions, lack of ingrowth into the components, aseptic-loosening, metallosis, necrosis, pseudotumors, and unexplained pain.^5,15^ While metallic debris particles are significantly smaller than polyethylene debris particles, they have been found to be more biologically active.^16^ ARMDs have been shown to be consistently higher in females,^17–19^ contributing to a nearly eightfold increase in MoM revision rates in women than men.^20,21^

A “pseudotumor” in this setting is a cystic lesion that is neither infective nor neoplastic that develops in the vicinity of a THA. Although the pathogenesis of ARMDs in general is not fully understood, histological analysis of pseudotumors and periarticular tissues of MoM THA shows a predominant lymphocyte immunologic response similar to a type IV hypersensitivity reaction.^22^ Pseudotumors have been reported in up to 69% of patients with MoM THA,^23^ with a higher incidence among women than men.^17,19,24^ The revision rate secondary to pseudotumor has been reported at 12%.^25^ While most pseudotumors are likely asymptomatic, symptoms associated with these lesions include hip mass, pain, limping, and rarely unilateral leg swelling. This case report describes the presentation, diagnostic workup, and treatment of a 58-year-old female with unilateral leg swelling due to an MoM pseudotumor. We also present a review of the existing case reports and literature on this rare complication.

## Case Report

A 58-year-old female presented for second opinion regarding her new-onset unilateral left lower extremity swelling, limited range of motion, pain, and hip “popping.” She had previously undergone right metal-on-polyethylene THA in 2002 and a left MoM THA in 2009. Her outside surgeon had ordered Doppler ultrasound to evaluate her swelling; this was negative for deep vein thrombosis (DVT). However, it showed large (7.5 × 4.0 cm) left hip effusion with mass effect on the adjacent vessels. Her medical history was significant for hypertension, type 2 diabetes, cardiomyopathy, congestive heart failure, and sarcoidosis. Her surgical history was positive for the aforementioned bilateral THAs as well as pacemaker placement, cardiac catheterization, hysterectomy, appendectomy, tonsillectomy, and adenoidectomy. The remainder of her history was unremarkable. On our initial examination, the patient had significant left lower extremity edema and fullness in her left groin. Femoral and peripheral pulses were palpable and equal bilaterally. She displayed an antalgic gait favoring her left side and painful range of motion of the left hip (20° passive internal and external rotation). Hip abduction strength was mildly decreased on the left compared with the contralateral side. There was no warmth, erythema, or other clinical signs of infection.

**FIG. 1.**
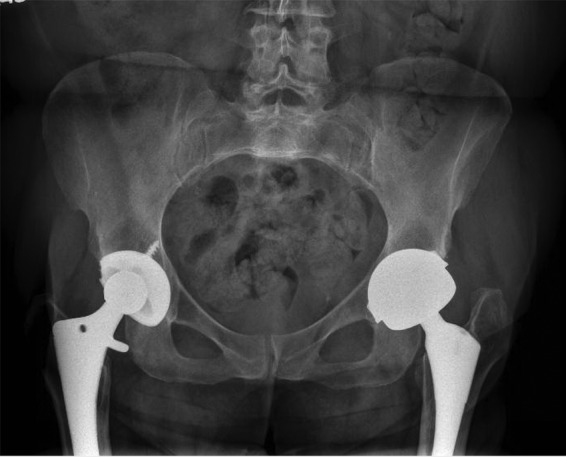
AP pelvis radiographs show right metal-on-polyethylene THA and left metal-on-metal THA. Components appear in appropriate positions without obvious signs of loosening. AP, anterior-posterior; THA, total hip arthroplasty.

Initial radiographs showed a well-positioned right THA with some eccentric wear of her polyethylene. The left hip radiographs showed an MoM arthroplasty with a well-fixed stem and the acetabular component in the appropriate position, without signs of loosening (Fig. 1). Initial workup included a computed tomography (CT) scan of the pelvis (the patient's pacemaker was not compatible with magnetic resonance imaging [MRI]); serum metal ion concentrations; laboratories, including C-reactive protein (CRP), erythrocyte sedimentation rate (ESR), and complete blood count (CBC); and a vascular surgery consult.

**FIG. 2.**
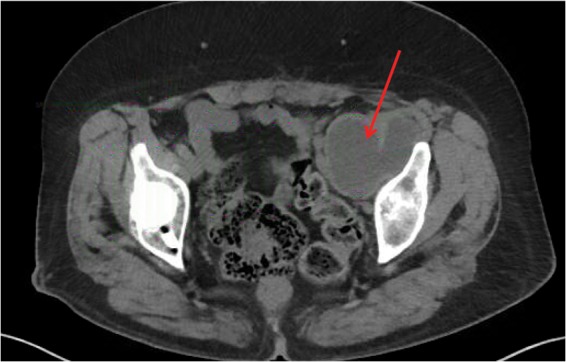
Axial cut from CT scan showing large fluid collection (arrow) measuring 5.5 × 6.0 × 10.4 cm that is originating from THA and displacing external iliac vasculature. CT, computed tomography.

Laboratory results were negative for infection, with normal CRP (0.27 (<1.0 mg/dL)), ESR (21 (0–30 mm/h)), and WBC (9.4 (4.5–11K/μL)). Cobalt and chromium metal ions were elevated at 15 (0–9 ng/mL) and 14 (<0.3 ng/mL), respectively. The noncontrast CT showed a large, simple fluid collection that extended into the pelvis and measured 5.5 × 6.0 × 10.4 cm (Fig. 2). The vascular surgery team repeated the ultrasound to again rule out DVT due to the patient's significant swelling. This ultrasound was again negative for DVT. After full review of the case, the vascular surgery team agreed that the size of the pseudotumor and compression on her external iliac vessels were the likely cause of her swelling and would be available for intraoperative assistance.

**FIG. 3.**
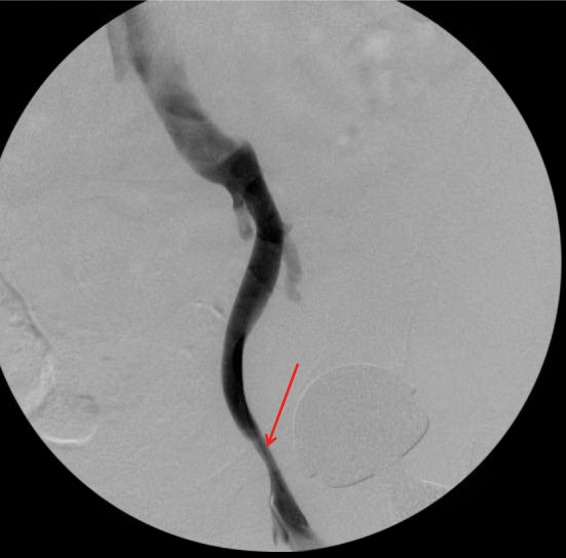
Venogram of external iliac vein performed intraoperatively by vascular surgery team showed ∼70% compression in area of pseudotumor (arrow).

**FIG. 4.**
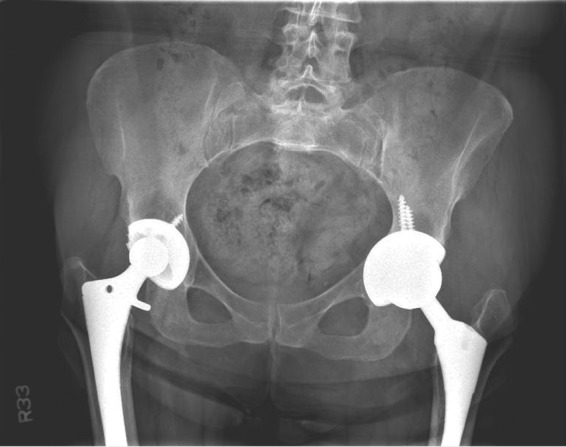
Revision left THA to metal-on-polyethylene construct.

After risks and benefits were discussed, the patient elected to proceed with surgery for revision left THA and pseudotumor decompression in a combination case with vascular surgery. Before incision, a fluoroscopic venogram showed ∼70% stenosis of the left external iliac vein without evidence of any thrombus. Vascular surgery deemed that no further intervention would be required at that time (Fig. 3). Revision THA was then performed via a standard posterior approach. A large quantity of dark brown, brackish fluid was found within a pseudocapsule (∼160 mL of fluid in total), which was completely excised. Intraoperative histopathology showed no signs of acute inflammation with <5 neutrophils per high powered field. Multiple cultures, including acid-fast, anaerobic/aerobic, and fungal were taken from both fluid and pseudocapsule. After thorough irrigation and debridement, the acetabular component was explanted and revised to a standard metal-and-polyethylene construct. The acetabular cup had very little evidence of bony ingrowth. The femoral component was well fixed, and thus, the original stem was retained and fitted with a metal femoral head (Fig. 4). Her postoperative course was uneventful, and she was discharged home on postoperative day 3. The intraoperative cultures remained negative and histopathology analysis showed “chronic inflammation and histiocytic reaction.”

The patient followed up with vascular surgery and orthopedic surgery ∼1 month postoperative and had complete resolution of her left lower extremity edema. Vascular surgery noted that no further intervention was indicated and that she could follow up on an as-needed basis. The patient was scheduled for follow-up in 6 months to 1 year with orthopedic surgery; however, she did not return to clinic until 4 years later. She had been doing very well in the interim, with pain-free ambulation without an assistive device and no recurrence of her lower leg edema.

## Discussion

Pseudotumors associated with MoM can present with a variety of symptoms; they have rarely been reported to cause unilateral leg swelling, as presented here. Our patient underwent evaluation for more common etiologies of swelling, such as infection and DVT. Imaging indicated that the swelling was due to compression of the external iliac vein by a pseudotumor originating from her MoM THA. Revision THA to a metal-on-polyethylene construct and excision of her pseudotumor resulted in complete resolution of her swelling by 1 month and lasted to her most recent follow-up, ∼4 years after surgery.

A review of the literature identified six published case reports that describe unilateral leg swelling after MoM THA.^26–31^ The result of each case is summarized in Table 1. Unfortunately, no other higher level studies or reviews are available; thus, no definitive statements can be made regarding patients with this condition. However, notable observations from review of these reports include the following: (1) 50% of the patients had an associated DVT, (2) 50% of the index procedures were MoM hip resurfacing, (3) 100% of patients required revision THA for resolution of their swelling, and (4) five of the six described patients were female. As with the current case, those in the previous case reports were initially evaluated for infection and DVT, and advanced imaging of the pseudotumor with either CT scan or metal artifact reduction sequence (MARS) MRI was obtained. In addition, intraoperative cultures, histopathology for acute inflammation, and permanent specimens were collected. All cultures were reported negative, and histopathology was consistent with adverse metal reaction ranging from macrophage giant cells with metal debris to perivascular lymphocyte predominant reactions. There was more variability among cases with respect to the use of serum metal ions, d-dimer, venogram, and hip aspiration for culture or metal ions before surgery.

**Table 1.** **Review and Summary of All Case Reports of Metal-on-Metal Pseudotumors Causing Unilateral Leg Swelling**

	Case reports with unilateral leg swelling associated with metal-on-metal pseudotumors				
	Patient demographics	Time from index procedure	Reported diagnostic workup	Intervention	Reported outcomes
Maurer-Ertl et al.^26^	38-year-old female	1 year (hip resurfacing)	X-ray, ultrasound (−DVT), CT scan, serum metal ions	Marginal resection followed by excision and revision ceramic-on-ceramic THA	Recurrence of swelling 10 months after marginal resection, complete resolution of swelling 20 months after revision THA
Parfitt et al.^27^	64-year-old male	1 year 4 months	X-ray, CRP, d-dimer, CBC, coagulation studies, ultrasound (+DVT), CT scan	IVC filter, thrombolysis, pseudotumor excision, and revision metal-on-polyethylene THA	Good functional recovery with resolution of groin discomfort
Algarni et al.^28^	54-year-old female	5 years	X-ray, ESR, CRP, ultrasound (−DVT), hip aspiration, aspirate metal ions, MRI	Pseudotumor excision and revision ceramic-on-ceramic THA	Complete resolution of swelling at 6 months, no recurrence at 1 year
Memon et al.^29^	54-year-old female	5 years (hip resurfacing)	X-ray, ESR, CRP, CBC, d-dimer, serum metal ions, ultrasound (+DVT), venogram, CT scan, hip aspiration, mass biopsy	IVC filter, anticoagulation, and revision ceramic-on-ceramic THA without excision	Significant improvement in swelling, occasional hip pain
Kawakita et al.^30^	69-year-old female	1 year 2 months	X-ray, ESR, CRP, d-dimer, ultrasound (−DVT), CT scan	Pseudotumor excision with subsequent revision ceramic-on-ceramic THA 1 year later	Decreased leg swelling 3 months after resection
Abdel-Hamid et al.^31^	75-year-old female	6 years (hip resurfacing)	X-ray, ESR, CRP, CBC, serum metal ions, ultrasound (+DVT), MRI	IVC filter, anticoagulation, vascular surgery-assisted excision and revision ceramic-on-polyethylene THA	Significant improvement in swelling 9 months after surgery

CBC, complete blood count; CRP, C-reactive protein; CT, computed tomography; DVT, deep vein thrombosis; ESR, erythrocyte sedimentation rate; IVC, inferior vena cava; MRI, magnetic resonance imaging; THA, total hip arthroplasty.

Maurer-Ertl et al. published the first report of an MoM pseudotumor causing unilateral leg swelling.^26^ The patient was a 38-year-old female who was 1 year from MoM hip resurfacing arthroplasty. The treating surgeon attempted marginal resection of the pseudotumor without revising the construct to a THA. The patient had recurrence of her symptoms ∼10 months later and subsequently underwent revision THA to a ceramic-on-ceramic construct with complete resolution of her swelling up to 20 months later. Parfitt et al. in 2012 published the first report of unilateral leg swelling related to a pseudotumor from an MoM THA; this is also the only case report published that described a male patient.^27^ As with the patients described by Memon et al.^29^ and Abdel-Hamid et al.,^31^ this patient was found to have a DVT and was treated with anticoagulation and inferior vena cava filter before surgical intervention for the pseudotumor. In addition, Abdel-Hamid et al. reported involving vascular surgery in the treatment paradigm. The vascular surgeons assisted in mobilization of the external iliac artery and vein and excision of the pseudotumor via a separate ilioinguinal approach.^31^

Our proposed diagnostic workup for patients with symptomatic MoM THA and ipsilateral leg swelling is similar to that published by Bolognesi and Ledford,^5^ with the addition of a vascular surgery consult and Doppler ultrasound to rule out DVT. As with THA of any type, complete workup of symptomatic MoM THA would include laboratory values to exclude infection, such as ESR, CRP, and CBC with differential. Additional laboratory workup should include serum metal ion levels. Imaging studies should include radiographic evaluation for proper component placement and signs of loosening, Doppler ultrasound for evaluation of DVT if clinically indicated, and advanced imaging with MARS MRI or CT scan to evaluate for osteolysis and pseudotumors. Consults should include vascular surgery if unilateral leg swelling is present and a pseudotumor is identified. Intraoperatively, we recommend sending tissue samples for culture, acute inflammation (neutrophils per high power field) and permanent sections for histopathology to assess for the presence of histiocytic reaction.

Based on our personal experience with this case, as well as our literature review, our proposed treatment for patients with unilateral leg swelling from MoM pseudotumor would be treatment of DVT if present, and combined surgical intervention with vascular surgery for excision of the pseudotumor. Revision THA should be performed to decrease the risk of recurrence of the pseudotumor. However, assessment of a larger series of patients is needed to be able to make recommendations with a higher level of evidence.

Women are uniquely impacted by MoM complications and demonstrate a significantly elevated revision rate than men.^20^ While the mechanism for this observed difference between males and females is not fully understood, differences in sex hormones, immune response, and environmental exposures to metal have been implicated.^32^ One proposed mechanism is a metal hypersensitivity reaction. Women have a higher rate of metal hypersensitivity than men, which may be associated with exposure to jewelry.^33^ MoM pseudotumors were initially termed aseptic lymphocyte-dominated vasculitis-associated lesions and have a histologic appearance and pathogenesis very similar to type IV hypersensitivity reactions.^22,34^ In addition, recent evidence shows that women demonstrate increased rates and severity of metal hypersensitivity after THA as measured by the lymphocyte stimulation index, which could be a source of unexplained pain in some patients.^32^ Thus, while MoM THA use has already dramatically decreased, we strongly recommend against the use of MoM constructs in females secondary to their increased metal hypersensitivity and increased risk for complications and need for additional surgeries.

## Conclusion

Pseudotumor is a known complication of MoM THA and can rarely cause unilateral leg swelling due to vascular compression. This swelling appears to be most reliably relieved with pseudotumor excision and revision THA. Patients should have a thorough workup to rule out DVT and infection, and we recommend involvement of vascular surgery in the evaluation and resection of pseudotumors. Women are at increased risk for complications from MoM THA, possibly related to metal hypersensitivity, and should be educated on these risks before surgery. If they elect to proceed with an MoM THA, they should be monitored closely for the development of complications.

## Abbreviations Used

ARMDs: adverse reactions to metal debris
CBC: complete blood count
CRP: C-reactive protein
CT: computed tomography
DVT: deep vein thrombosis
ESR: erythrocyte sedimentation rate
MARS: metal artifact reduction sequence
MoM: metal-on-metal
MRI: magnetic resonance imaging
THA: total hip arthroplasty
